# Sol-Gel Synthesis of Zinc Alumotitanate, Monitoring of Chelation, Hydrolysis, Condensation, and Crystallization Processes

**DOI:** 10.3390/molecules29050965

**Published:** 2024-02-22

**Authors:** Stanislav Kurajica, Vilko Mandić

**Affiliations:** Faculty of Chemical Engineering and Technology, University of Zagreb, Marulićev trg 19, HR 10000 Zagreb, Croatia; vmandic@fkit.unizg.hr

**Keywords:** chelate, ethyl acetoacetate, sol-gel, NMR spectroscopy

## Abstract

Zinc alumotitanate sorbents with various compositions were prepared through sol-gel synthesis with the use of ethyl acetoacetate as a chelating agent. The formation and decomposition of chelates, providing insight into sol-gel process advancement, have been successfully monitored via ^1^H NMR, ^13^C NMR, and FTIR spectroscopy. It has been established that Al(OBu^s^)_3_ and Ti(OBu^n^)_4_ react completely with Eaa, forming chelates after 1 h, while after 24 h hydrolysis is already advanced. Hydrolysis is accelerated in the presence of Zn(NO_2_)_3_·6H_2_O, supplying the water needed for hydrolysis. In dried gels, the amount of ethyl acetoacetate is greatly reduced, and it is mainly present unbound. According to XRD analysis, samples with none or less titania are composed of layered double hydroxide, while in samples with greater amounts of titania, crystal nitrates are present. In all samples except those without Al, the spinel phase with variable composition crystallizes.

## 1. Introduction

Integrated Gasification Combined Cycle (IGCC) enables increased efficiency of energy production through the use of a steam and gas turbine. However, in order to avoid equipment corrosion, the process requires hydrogen sulfide to be separated from hot gases. For this purpose, high-temperature gas desulfurization (HTGD) is utilized. The process consists of sulfur sorption into metal oxide (sorbent) and the regeneration of sorbents by oxidation. The sorbent must be chemically, thermally, and mechanically stable to withstand numerous cycles of sorption and regeneration. Numerous metal oxides have been tested as sorbents for the desulfurization process [1]. Zinc oxide-based sorbents have been found to be the most effective [2]. Unfortunately, under conditions prevailing in the desulfurization process, Zn^2+^ tends to reduce to elemental zinc, and at the high temperatures at which the process takes place, elemental zinc evaporates. This process is the primary cause of the decrease in sorbent capacity after several successive cycles of desulfurization and regeneration [3]. Also, since regeneration takes place in a fluidized bed reactor, significant mechanical wear of the sorbent occurs, and high process temperatures promote the sintering of the metal oxide, which reduces its active surface [3]. In order to avoid those shortcomings, ZnO was combined with other metal oxides [4,5,6], where TiO_2_ yielded the best results, preventing zinc reduction and evaporation [7]. Remaining problems such as a tendency for mechanical wear and sintering can be solved by the addition of high-temperature stable metal oxides such as Al_2_O_3_ [8,9]. Al_2_O_3_ should also have a beneficial effect on the pore structure and increase the specific surface area of zinc-titanate-based sorbents, facilitating the interaction of gas and solid during the process of sulfation and sorbent regeneration [9].

In contrast to the intensive investigation of the most favorable chemical and phase composition of sorbents, few researchers have noted the importance of the preparation process. Common methods of sorbent preparation are the solid-state reaction of previously finely ground and mixed oxides or precipitates obtained by coprecipitation [10]. These techniques require the use of very high temperatures to allow the reaction to be solid-state, which in turn results in a reduction of the specific surface area and a loss of reactivity [11]. A more efficient synthesis method is the sol-gel process since it allows better precursor mixing, lower sintering temperatures, and greater product homogeneity [12]. Therefore, the prepared sorbent would be characterized by a larger specific surface area and a balanced phase composition. The sol-gel process consists of the hydrolysis of precursors and the polycondensation of hydrolyzed molecules to form a colloidal sol. Further polymerization results in the transition of the liquid salt to the solid gel phase. Common precursors for sol-gel synthesis are metal alkoxides, but their frequent drawback is rapid hydrolysis, resulting in uncontrolled hydroxide deposition [13]. In order to prevent uncontrolled rapid hydrolysis, alkoxide precursors are modified by the addition of chelating agents. Chelating agents replace alkoxyl groups, which increases hydrolytic stability and alters the course of condensation reactions. In the manner described, in addition to establishing better control over the hydrolysis and condensation processes, the final properties of the obtained materials, such as phase composition, microstructure, texture, and physical properties, can be affected [13]. Commonly used chelating agents are β-ketoesters, bidentate ligands with a single negative charge. The coordination of β-ketoesters with aluminum or titanium atoms creates a complex in which both oxygen atoms are bonded to a metal atom. In addition to chelating agents, the final properties of the material can be influenced by the control of stoichiometry, process conditions, drying and aging of the gel, and thermal treatment. Ethyl acetoacetate is less commonly used as a chelating agent, so the chelation of aluminum and titanium alkoxides with ethyl acetoacetate has been poorly studied.

Therefore, in this investigation, a precursor for ZnO-Al_2_O_3_-TiO_2_ sorbent was prepared through sol-gel synthesis with the use of ethyl acetoacetate as the chelating agent. Sols with various compositions, aiming at element ratios enabling the formation of a solid solution with the spinel structure, were prepared and investigated using ^1^H NMR and ^13^C NMR spectroscopy. Gels were examined via FTIR, while dried and thermally treated gels were characterized by XRD.

## 2. Results and Discussion

**Figure 1:**
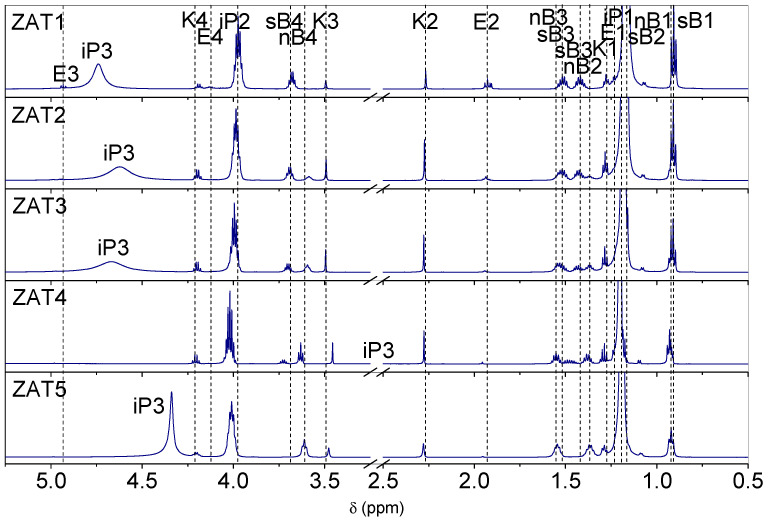
^1^H NMR spectra of investigated samples after 24 h of hydrolysis.

**Figure 2:**
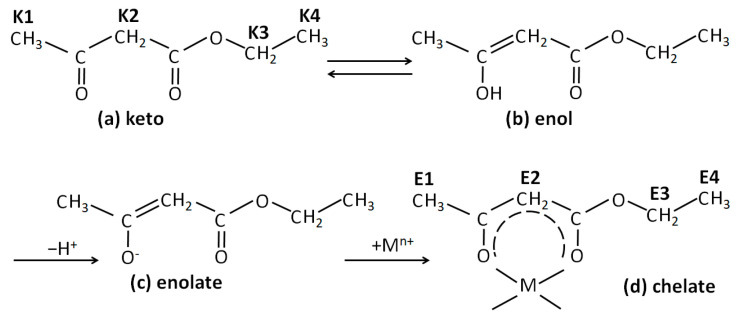
Schematic representations of the structure of the molecules of (**a**) keto tautomer of Eaa, (**b**) enol tautomer of Eaa, (**c**) enolate of Eaa, and (**d**) metal-Eaa chelate.

^1^H NMR spectra of samples ZAT1-ZAT5 after 24 h of hydrolysis are presented in Figure 1. Hydrogen atoms are labeled according to Figure 2. Assignments of spectra were performed on the basis of chemical shifts, signal intensities, magnitude and multiplicity, NMR spectra of pure precursors, chelating agents, and solvents, as well as on the basis of previous findings [12,13,14]. The most pronounced resonances in the spectra are those due to isopropanol at ~1.19 ppm for methyl protons (iP1), ~3.99 ppm for methine protons (iP2), and signals centered at 4.74 (ZAT1), 4.62 (ZAT2), 4.67 (ZAT3), 2.75 (ZAT4), and 4.34 (ZAT5) due to the hydroxyl group protons. The characteristic resonances of sec-butoxy groups of AsB occur at ~0.90 and ~1.16 (sB2) ppm for methyl protons, at ~1.42 and ~1.51 ppm for methylene protons, and at ~3.70 for methyne protons. The characteristic resonances of the n-butoxide groups of TnB occur at ~0.92 ppm for methyl protons and at ~1.37 ppm, ~1.55 ppm, and ~3.59 ppm for methylene protons. The intensities of signals are in concordance with the stoichiometry of the samples: the resonances of sec-butoxy groups of AsB are the most intense in the spectra of sample ZAT1, diminish in the spectra of sample ZAT4, and do not occur in the spectra of sample ZAT5. Of course, it is quite the opposite for the resonances of n-butoxy groups of TnB (most intense in the spectra of sample ZAT5 and non-existent in the spectra of sample ZAT1). No resonances due to sec-butanol and n-butanol released from alkoxides in the course of the chelation process could be observed.

Resonances of Eaa could also be observed in the spectra of the investigated samples. Eaa is a β-ketoester capable of undergoing keto-enol tautomerism (Figure 2a,b). In pure Eaa, the ketonic form predominates, while in the presence of metal alkoxide, the enol form is deprotonated, and the obtained enolate substitutes the alkoxy groups of alkoxide (Figure 2c), forming a chelate (Figure 2d). Replacement of the alkoxy group with the enolate, i.e., the formation of a chelate, strongly shifts the keto-enoli equilibrium toward the enol tautomer. In contrast, hydrolysis causes the release of Eaa, and the shifting of the keto-enolic equilibrium back towards the keto tautomer can be observed. The ^1^H NMR spectra of the samples exhibited resonances for keto tautomer methyl protons at ~1.27 ppm (K1) and at ~2.28 ppm (K2), and for methylene protons at ~3.50 ppm (K3) and ~4.20 ppm (K4). The characteristic signals of the enolate Eaa in the chelate occur at ~1.24 ppm (E1) and ~1.93 (E2) for methyl protons, at ~4.14 for methylene (E4) protons, and at ~4.93 for methyne (E3) protons.

All of the spectra show the presence of Eaa in both, ketonic and enolate forms. This comes as a surprise since, in the preliminary investigation, a complete chelation after 24 h was observed, i.e., no ketonic form of Eaa was observed.

**Figure 3:**
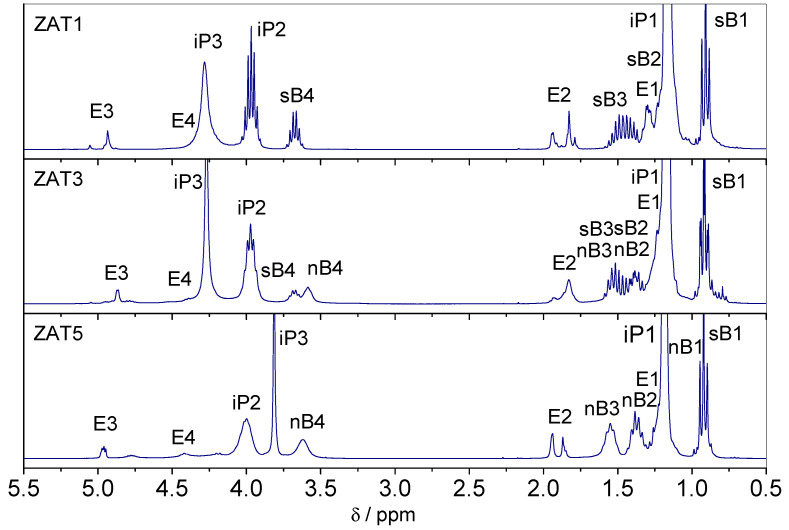
^1^H NMR spectra of samples ZAT1, ZAT3, and ZAT5 after 1 h of hydrolysis.

In order to clarify this inconsistency, the state of the system 1 h after mixing was checked with ^1^H NMR (Figure 3). It turned out that after 1 h, the chelation was complete since spectra show no resonances due to Eaa in keto form. Therefore, after 1 h, samples are completely chelated, while after 24 h, the hydrolysis could be rated as advanced. Obviously, the presence of water introduced through zinc nitrate hexahydrate accelerated the hydrolysis process. The influence of water on the hydrolysis rate is self-explanatory and well known [15]. The ratio between ketonic Eaa and enolate Eaa in samples after 24 h could be obtained by the integration of K2 and E2 resonances in Figure 1. The integration yields 37:63, 78:22, 89:11, 92:8, and 95:5 for samples ZAT1, ZAT2, ZAT3, ZAT4, and ZAT5, respectively. Also, it is obvious that the overall Eaa content, keto and enolic, in the samples decreases from ZAT1 to ZAT5, which is a consequence of β-ketoester evaporation. Also, the decrease in the butoxide groups’ amount is observed in the same order. It is evident that the extent of hydrolysis increases from sample ZAT1 to sample ZAT5. Two reasons for that could be noted: Firstly, the amount of water increases from sample ZAT1 to sample ZAT5, and consequently, the hydrolysis and condensation rates increase. Secondly, the hydrolysis and condensation rates depend on the electronegativity of the metal atom. The metal with lower electronegativity undergoes hydrolysis more rapidly than the one with higher electronegativity [15]. The difference between electronegativity between Ti and Al is not great, but Ti indeed has lower electronegativity (1.5) than Al (1.6) [16]. Therefore, it could be argued that aluminum chelate is more stable than titanium chelate. However, since hydrolysis was not observed in samples without Zn(NO_3_)_2_·6H_2_O after 24 h of synthesis in a closed reactor, it is obvious that the increased amount of water is the primary reason for advanced hydrolysis.

Multiple sets of enolate Eaa resonances could be observed, which is the consequence of the presence of ineqivalent chelating sites [12,14]. Structural models proposed for AsB and TnB chelated with β-diketones [12,14] suggest that such compounds are oligomers and isomers possessing different chelation sites, giving rise to multiple resonances.

**Figure 4:**
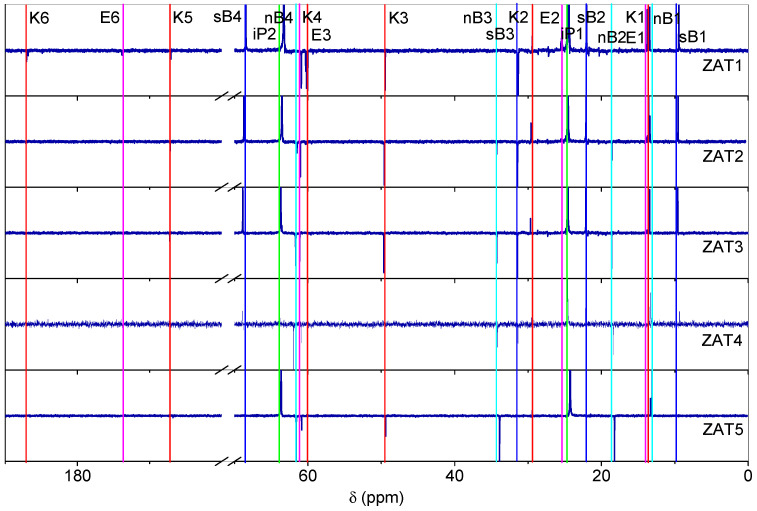
^13^C NMR spectra of investigated samples after 24 h of hydrolysis.

The ^13^C NMR spectra of the investigated samples (Figure 4) are in accordance with the ^1^H NMR spectra. The signals due to the enolic form of Eaa bonded to Al or Ti were as follows [12,13,14]: methyl carbons at 13.7 ppm (E1) and 25.4 ppm (E2), methylene carbon at 60.6 ppm (E4), methyne carbon should be around 88 ppm (E3), but it was not observed, and quaternary carbon at 173.7 ppm (E5), another quarternary carbon should be around 184 ppm (E6), but it was also not observed. On the other hand, all signals of the free keto form were noted: K1 at 13.9 ppm, K2 at 29.5 ppm, K3 at 49.5 ppm, K4 at 61.1 ppm, K5 at 167.2 ppm, and K6 at 187.0 ppm. Signals due to both forms decrease in intensity from ZAT1 to ZAT5 samples. The ^13^C NMR spectroscopy confirmed that the majority of Eaa was present in the keto form. The ^13^C NMR spectra are also characterized by the expected resonance signals of sec-butoxide: sB1 at 9.5 ppm, sB2 at 22.0 ppm, sB3 at 31.6 ppm, and sB4 at 68.5 ppm; n-butoxide: nB1 at 13.1 ppm, nB2 at 18.7 ppm, nB3 at 34.4 ppm, and nB4 at 61.8 ppm; and isopropanol: iP1 24.62 and iP2 64.0 ppm. The signal due to CDCl_3_ appears at 76.5 ppm.

**Figure 5:**
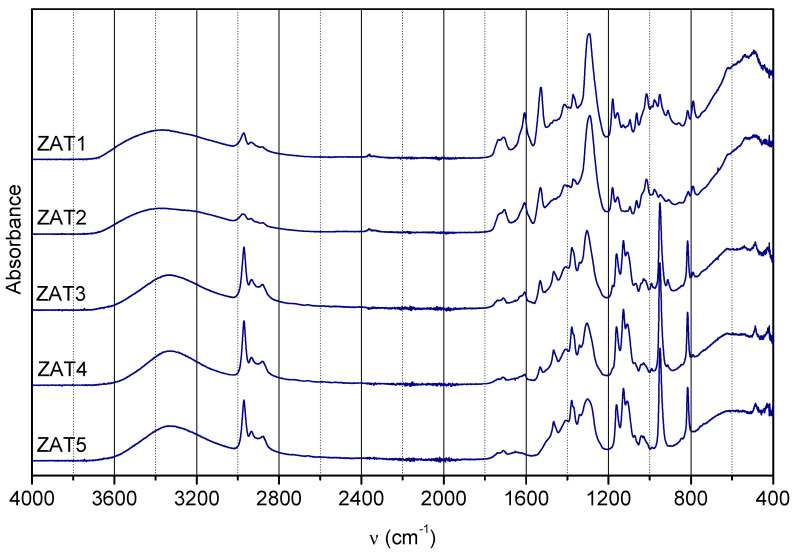
FTIR spectra of investigated samples 7 days after synthesis.

In order to gain information on gel composition, the FTIR spectra of samples ZAT1-5 after gelation were acquired (Figure 5). As already mentioned, Eaa could be present as keto and enol tautomers, as well as enolate bonded in chelate. For keto tautomer, the most characteristic bands in FTIR spectra occur at 1735 and 1710 cm^−1^ (C=O stretching vibrations of two carbonyl groups); for enol tautomers, most characteristic bands are at ~1650 cm^−1^ (hydrogen bonding between the ester C=O and the enolic hydroxyl group) and ~1630 cm^−1^ (alkene bond in conjugation with a carbonyl group), while for enolate bonded in chelate, most characteristic bands are ~1610 cm^−1^ (C–O bond in enolate bonded to Al) and ~1530 cm^−1^ (C–C vibration of the six-membered complex ring) [12,13,14]. As can be observed, the amount of Eaa, regardless of form, decreases from sample ZAT 1 to ZAT 5. Also, the keto-to-enolate ratio increases from sample ZAT 1 to ZAT 5. Both observations are in concordance with ^1^H NMR findings for sols after 24 h of hydrolysis.

Other bands common to both tautomers and enolates are as follows: 2980 cm^−1^ (asymmetrical C–H stretching in CH_3_ groups), 2870 cm^−1^ (symmetrical C–H stretching in CH_3_ groups), 2940 cm^−1^ (asymmetrical C–H stretching in CH_2_ groups), 2850 cm^−1^ (symmetrical C–H stretching in CH_2_ groups), 1466 cm^−1^ (asymmetrical C–H bending vibration in CH_3_ groups), 1370 cm^−1^ (symmetrical bending vibration in CH_3_ groups), 1470 cm^−1^ (scissoring band of CH_2_) a few bands in the 1350–1150 cm^−1^ region (methylene twisting and wagging vibrations in esters), 1013 cm^−1^ (O–C–C stretching), 1178 cm^−1^ (–C–C–O stretching) and 1290 cm^−1^ (C–C stretching), 1415 cm^−1^ (C–H rocking vibration of alkene) [12,14,17,18].

Literature is scarce on the 1128, 816, and 948 cm^−1^ bands in this system. According to Farber et al. [19], the first could be due to C-O stretching in alcohols, while the latter two can be attributed to Zn-O stretching [20]. However, the attribution of these bands remains an open question. The broad band in the range 400–800 cm^−1^ is due to Zn-O, Ti-O, and Al-O bonds, as well as O-M-O bonds in brucite-like layers [21]. In this range are also bands typical for LDH [22,23], while the broad band between 3600 and 3000 cm^−1^ is due to OH stretching vibrations [24].

**Figure 6:**
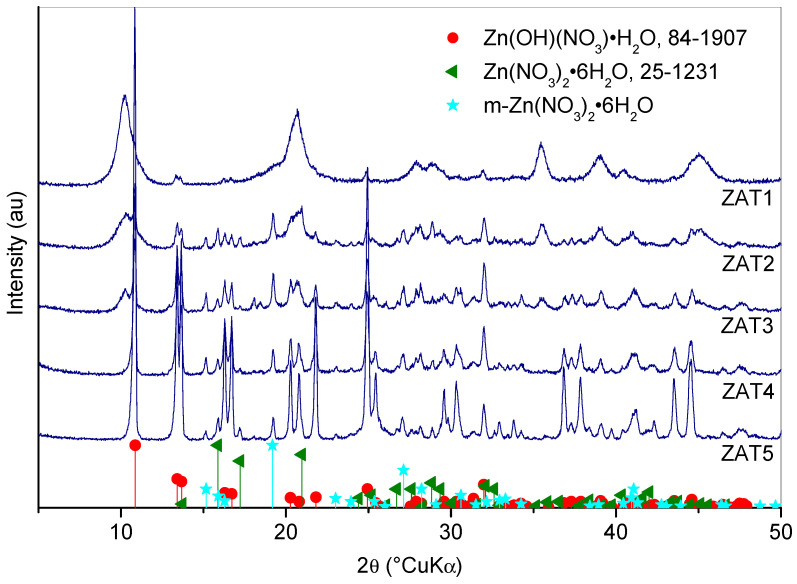
XRD patterns of investigated gel samples 7 days after synthesis.

XRD patterns of dried gels are given in Figure 6: The XRD pattern of sample ZAT1, and to a lesser extent, ZAT2 and ZAT3, exhibits several broad peaks attributed to the layered double hydroxide (LDH) phase [23,25]. LDH is a compound composed of a positively charged brucite-like layer with negatively charged balancing ions in the interlayer [26]. The pattern is characterized by two distinctive peaks at low angles corresponding to the basal (003) and (006) LDH reflections. The LDH lattice parameter c corresponds to a triple value of d_003_. Based on reflection d_003_ = 8.61 Å, the unit cell parameter c was calculated to be 25.83 Å [25]. For a similar system comprising different shares of aluminum and titanium, Santamaria et al. [25] report a parameter c of about 22.7 Å. However, the introduction of organic molecules yields a considerable interlayer increase. Therefore, we conclude that brucite-type layers comprise cations, while NO_3_^−^, water, and organic molecules are in the interlayer space [25,27]. The decrease in LDH peaks intensity from sample ZAT1 to sample ZAT3, and the absence of these peaks in samples ZAT4 and ZAT5, is probably a consequence of greater distortions in samples with a greater titanium share. Santamaria et al. [25] also noted the decrease in LDH crystallinity with the increase in titanium content and considered this an indication of the incorporation of titanium into LDH. On the XRD pattern of sample ZAT2, peaks of Zn(OH)(NO_3_)·H_2_O (ICDD PDF #84-1907) appear and grow stronger toward sample ZAT5. In the diffraction pattern of sample ZAT5, all the diffraction peaks of this phase are evidently present, the most prominent of which are (200), (202), and (002) at 10.86, 13.41, and 13.67 °2θ, respectively. Two additional sets of peaks appear in the XRD pattern. One is due to orthorhombic Zn(NO_3_)_2_·6H_2_O (ICDD PDF #25-1231); this phase is present in a much smaller proportion, so its diffraction peaks are significantly smaller than the peaks of Zn(OH)(NO_3_)·H_2_O. Some of the Zn(NO_3_)_2_·6H_2_O peaks are also overlapped with the peaks of the dominant phase. However, the diffraction peaks at 15.87, 17.22, 26.70, and 28.82 °2θ from the reflexes (210), (111), (311), and (400), respectively, are clearly visible, making the presence of this phase beyond any doubt. The other set of peaks agrees best with α-Co(NO_3_)_2_·6H_2_O (ICDD PDF #25-1219). Of course, there is no cobalt in the sample, and we can only assume that these peaks are due to a phase with the same structure but altered chemical composition. Based on the same oxidation state and relatively close radii of Co^2+^ and Zn^2+^, the Zn(NO_3_)_2_·6H_2_O phase seems to be the most likely. As α-Co(NO_3_)_2_·6H_2_O has a monoclinic structure, this phase is denoted as m-Zn(NO_3_)_2_·6H_2_O. Unfortunately, we could not find diffraction data for such a phase, so it cannot be positively identified. Judging by the appearance of the diffraction patterns and the lack of peaks of phases containing aluminum and titanium, it is clear that some parts of the samples are amorphous.

**Figure 7:**
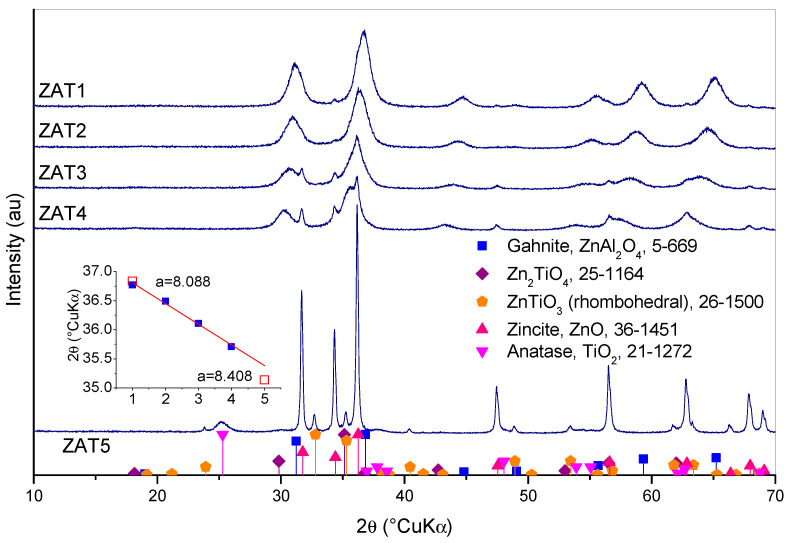
XRD patterns of investigated gel samples thermally treated at 500 °C for 2 h.

Diffraction patterns of samples thermally treated for 2 h at 500 °C are shown in Figure 7. Sample ZAT1 is composed of gahnite, ZnAl_2_O_4_ (ICDD PDF #5-0669), and traces of zincite, ZnO (ICDD PDF #36-1451). The same is true with sample ZAT2, but a shift in gahnite diffraction peaks could be observed, and zincite peak intensity increased, although it could still be rated as weak. The shift of gahnite peaks points out the solid solution formation, i.e., the entrance of Ti in the crystal lattice of ZnAl_2_O_4_. An even greater shift of gahnite peaks could be observed in the diffraction patterns of samples ZAT3 and ZAT4. The inset in Figure 7 shows spinel (311) peak positions for samples ZAT1 to ZAT4. Positions of (311) peaks for ZnAl_2_O_4_ according to ICDD PDF #5-0669 and Zn_2_TiO_4_ according to ICDD PFD #25-1164 are also shown (hollow squares). Both, ZnAl_2_O_4_ and Zn_2_TiO_4_, have the same spinel structure, so the variation in (311) peak position is the consequence of lattice constant change due to compositional alterations. Lattice constants for pure gahnite and pure ZnTiO_3_ are also shown for comparison. As can be observed, the (311) peak’s shift roughly agrees with the change in spinel composition from pure ZnAl_2_O_4_ in sample ZAT1 to Ti-rich spinel in sample ZAT4. However, a composition equal to Zn_2_TiO_4_ is not reached in sample ZAT5. In fact, the diffraction pattern of sample ZAT5 is rather different than the other four patterns. Here, well-crystallized zincite, whose peaks were growing in intensity from sample ZAT1 to ZAT4, is the main phase. Instead of cubic Zn_2_TiO_4_, a rhombohedral ZnTiO_3_ (ICFF PDF #26-1500) is present, and anatase TiO_2_ (ICDD PDF #21-1272 TiO_2_) appears.

**Figure 8:**
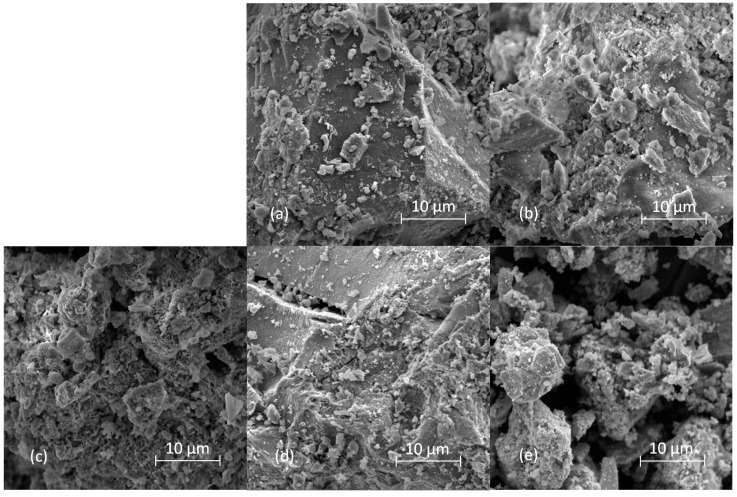
SEM micrographs of investigated samples thermally treated at 500 °C for 2 h. (**a**) ZAT1, (**b**) ZAT2, (**c**) ZAT3, (**d**) ZAT4, and (**e**) ZAT5.

Scanning electron microscope micrographs of samples thermally treated for 2 h at 500 °C are shown in Figure 8. All samples but ZAT 5 are composed of polydisperse particles with irregular shapes and size distributions. For some of the micrographs, e.g., Figure 8b,c, it can even be said that particles with plate morphology are observed on them. The retention of the original LDH plate morphology after calcination was also reported by other authors [25]. In the micrograph of sample ZAT5, only heavily agglomerated, smaller particles could be observed. It could be said that the difference in ZAT5 sample morphology in comparison with the other samples is consistent with the difference in the phase composition of this sample compared to the others.

Since the prepared material is intended to be a sulfur sorbent, the following research will include sorbent sulfidation performance. The prepared materials will be subjected to the sulfidation process, followed by thermo-gravimetric and phase analysis. Sulfidation reactivity and stability will be tested in order to select the most suitable sorbent for sulfur removal.

## 3. Materials and Methods

For the synthesis, the following precursors were used: aluminium sec-butoxide (Al(OsBu)_3_, 97%, Acros organics, Geel, Belgium), denoted AsB, titanium n-butoxide (Ti(OnBu)_4_, 98%, Alfa Aesar, Karlsruhe, Germany), denoted TnB, zinc nitrate hexahydrate (Zn(NO_3_)_2_·6H_2_O, 98%, Riedel de Haën, Berlin, Germany), denoted ZnN. Ethyl acetoacetate (C_6_H_10_O_3_, EAA, 99%, Fluka, Buchs, Switzerland), denoted Eaa, was used as a chelating agent, and isopropyl alcohol (C_3_H_7_OH, IPA, 99%, Carlo Erba Reagents, Cornaredo, Italy), denoted iP, was used as a solvent.

During preliminary investigations, it was established that Eaa reacts completely with AsB and TnB, forming chelate, while ZnN did not react with Eaa at all. Also, it was established that the optimal molar ratio of AsB and TnB to Eaa is 1:1. Therefore, the synthesis procedure started with the preparation of three separate solutions in iP: AsB + Eaa, TnB + Eaa, and ZnN. For the preparation of the AsB+Eaa solution, Eaa was mixed with iP in a molar ratio of 1:10, and then AsB in the same molar amount as Eaa was added using a syringe to minimize exposure to atmospheric humidity. The other two solutions were prepared in the same manner and in the same molar amount, except in the preparation of the ZnN solution, Eaa was omitted. The mixtures were stirred for 30 min, then the TnB + Eaa + iP mixture was slowly added to the AsB + Eaa + iP mixture in the desired stoichiometry, stirred for another 30 min, and then the ZnN + iP mixture was added. Five samples were prepared, ZAT1 to ZAT5; their compositions are given in Table 1. Those compositions were selected since they lay at the straight line between ZnAl_2_O_4_ and Zn_2_TiO_4_ in the ZnO-Al_2_O_3_-TiO_2_ ternary diagram. In this way, samples having a composition favorable for the formation of a solid solution with a spinel structure were prepared. The mixtures were stirred for 24 h at room temperature. The obtained samples were dried in the air, ground to a fine powder, and stored in a closed container. Part of the samples was thermally treated at a temperature of 500 °C for 2 h. This temperature was chosen because it was established that after heat treatment at this temperature, the mass of the samples remains nearly constant.

^1^H and ^13^C NMR spectra were recorded in NMR Spectrometer Bruker Avance 300 NMR Spectrometer, Billerica, USA, operating at 300 MHz for ^1^H resonance and for ^13^C at 75 MHz. As a solvent and internal standard, CDCl_3_ and Si(CH_3_)_4_ were used, respectively. The 5 mm NMR tubes were used for measurement. The ^1^H and ^13^C NMR chemical shift values (δ) are expressed in ppm, referring to Si(CH_3_)_4_. IR spectra were acquired using the Fourier transform infrared (FTIR) spectrometer Bruker Vertex 70, Billerica, USA, in ATR (attenuated total reflectance) mode. The samples were pressed on a diamond, and the absorbance data were collected between 400 and 4000 cm^−1^ with a spectral resolution of 1 cm^−1^ and 64 scans. XRD patterns were obtained using the Shimadzu diffractometer XRD 6000, Kyoto, Japan, with CuKα radiation. Data were collected between 5 and 70° 2θ in a step scan mode with steps of 0.02° and a scan speed of 0.03 °2θ s^−1^. SEM micrographs were obtained using the scanning electron microscope Tescan Vega EasyProbe3, Brno, Czechia, in secondary electron mode. Powders were put on adhesive carbon tape and sputtered with palladium/gold on Sputter Coater SC7620 for 45 s at 18 mA in Ar plasma.

## 4. Conclusions

Chelation, hydrolysis, and condensation processes in the course of the sol-gel synthesis of zinc alumotitanate were monitored using ^1^H NMR, ^13^C NMR, and FTIR spectroscopy. Information on processes’ advancement was gained on the basis of the appearance of keto, enol, and enolate characteristic resonances. It was established that Al(OBu^s^)_3_ and Ti(OBu^n^)_4_ react completely with Eaa, forming chelates after 1 h. After 24 h, the majority of Eaa is present as keto tautomer. The decomposition of the chelate points out advanced hydrolysis and condensation, i.e., the progress of the sol-gel process. Hydrolysis is accelerated in the presence of Zn(NO_2_)_3_·6H_2_O, which supplies the water needed for hydrolysis. In dried gels, the amount of ethyl acetoacetate is greatly reduced; ethyl acetoacetate is mainly present as keto tautomer and thus unbound. Samples with none or less titania are composed of layered double hydroxide, while in samples with a greater amount of titania, crystal nitrates are present. In all samples except those without Al, the spinel phase with variable composition crystallizes.

## Figures and Tables

**Table 1:** Sample compositions expressed according to molar ratios of oxides.

Sample	ZAT 1	ZAT 2	ZAT 3	ZAT 4	ZAT 5
ZnO	0.500	0.556	0.600	0.636	0.667
Al_2_O_3_	0.500	0.333	0.200	0.091	0
TiO_2_	0	0.111	0.200	0.273	0.333

## Data Availability

The raw data supporting the conclusions of this article will be made available by the authors on request.
